# Мониторинг эффективности программы профилактики заболеваний, связанных с дефицитом йода в Республике Тыва

**DOI:** 10.14341/probl12715

**Published:** 2021-01-28

**Authors:** Е. А. Трошина, Н. В. Мазурина, Е. С. Сенюшкина, Н. П. Маколина, М. О. Галиева, Л. В. Никанкина, Н. М. Малышева, А. Б. Даржаа, Ю. С. Сенги

**Affiliations:** Национальный медицинский исследовательский центр эндокринологии; Национальный медицинский исследовательский центр эндокринологии; Национальный медицинский исследовательский центр эндокринологии; Национальный медицинский исследовательский центр эндокринологии; Национальный медицинский исследовательский центр эндокринологии; Национальный медицинский исследовательский центр эндокринологии; Национальный медицинский исследовательский центр эндокринологии; Министерство здравоохранения Республики Тыва; Министерство здравоохранения Республики Тыва

**Keywords:** йодный дефицит, диффузный зоб, йодированная соль

## Abstract

**Обоснование:**

Обоснование. Республика Тыва — регион с доказанным тяжелым природным йодным дефицитом и высокой распространенностью ЙДЗ (йододефицитных заболеваний). Однако в регионе в определенные периоды времени предпринимались меры по ликвидации дефицита йода в питании населения. В статье представлены результаты проведенного в октябре 2020 г. специалистами ФГБУ «НМИЦ эндокринологии» Минздрава России контрольно-эпидемиологического исследования, направленного на оценку современного состояния йодной обеспеченности населения Республики Тыва. Исследование проведено по поручению Министерства здравоохранения России в рамках государственного задания «Научная оценка необходимости принятия дополнительных нормативных правовых и иных мер по ликвидации йодного дефицита в пилотных регионах с тяжелым йодным дефицитом» (фрагмент, посвященный контрольно-эпидемиологическим исследованиям в регионе), «Эпидемиологические и молекулярно-клеточные характеристики опухолевых, аутоиммунных и йододефицитных тиреопатий как основа профилактики осложнений и персонализации лечения» (аналитический обзор мероприятий, предшествующих контрольно-эпидемиологическим исследованиям в регионе).

**Цель:**

Цель. Оценка йодной обеспеченности населения Республики Тыва.

**Материалы и методы:**

Материалы и методы. Исследование проводилось в трех населенных пунктах республики — гг. Кызыл, ­Шагонар, п. Сарыг-Сеп. Обследованы 227 школьников допубертатного возраста (8–10 лет) с проведением сбора анамнеза, осмотра врача-эндокринолога, пальпации щитовидной железы, забора разовых образцов мочи в одноразовые микропробирки типа Эппендорф, с последующей заморозкой до -20–25 °С для дальнейшего определения концентрации йода в моче с помощью церий-арсенитного метода в лаборатории (клинико-диагностическая лаборатория ФГБУ «НМИЦ эндокринологии» Минздрава России). Кроме того, всем школьникам выполнено ультразвуковое исследование щитовидной железы (с использованием портативного ультразвукового аппарата LOGIQe (China) с мультичастотным линейным датчиком 10–15 МГц, в положении лежа). Рост и вес детей определялись по стандартной методике в момент обследования. Проведены сбор образцов пищевой соли, которая используется в семьях школьников, и определение наличия йодата калия в ней экспресс-методом.

Родители школьников подписали информированные согласия на проведение обследования детей. Разрешение локального этического комитета ФГБУ «НМИЦ эндокринологии» Минздрава России получено 25 марта 2020 г., № 5.

**Результаты:**

Результаты. Обследованы 227 школьников 8–10 лет. Определена медианная концентрация йода в моче, исследовано наличие йодата калия в пищевой соли и проведено ультразвуковое исследование щитовидной железы с целью уточнения йодной обеспеченности, охвата использования йодированной соли в питании и распространенности зоба.

Медианная концентрация йода в моче составила 153 мкг/л, частота зоба — 7,7%, доля йодированной соли — 95,2%.

**Заключение:**

Заключение. Показатель медианной концентрации йода в моче свидетельствует об оптимальной йодной обеспеченности ­населения Тывы. Частота зоба у школьников значимо снизилась по сравнению с данными, полученными в ходе предыдущих эпидемиологических исследований. Доля домохозяйств, использующих йодированную соль (­более 90%), свидетельствует об эффективности ­профилактических мероприятий.

## Abstract (en)

**Background:**

Background: The Republic of Tyva is a region with a proven severe natural iodine deficiency and a high prevalence of IDD (iodine deficiency disorders). However, in the region in certain periods of time, measures were taken to eliminate iodine deficiency in the diet of the population. The article presents the results of the October 2020. by specialists of the Endocrinology Research Centre, a control and epidemiological study aimed at assessing the current state of iodine supply in the population of the Republic of Tyva. The study was carried out on behalf of the Ministry of Health of Russia within the framework of the state task «Scientific assessment of the need to take additional regulatory legal and other measures to eliminate iodine deficiency in pilot regions with severe iodine deficiency».

**Aim:**

Aim: Assessment of iodine supply of the population of the Republic of Tuva.

**Materials and methods:**

Materials and methods: The research was carried out in three settlements of the republic — years. Kyzyl, Shagonar, Saryg-Sep settlement. A total of 227 pre-pubertal schoolchildren (8–10 years old) were examined with anamnesis collection, examination by an endocrinologist, palpation of the thyroid gland, collection of single urine samples into disposable eppendorfs, followed by freezing to minus 20–25 degrees to further determine the concentration of iodine in urine with using the cerium-arsenitic method in the laboratory (clinical diagnostic laboratory of the Federal State Budgetary Institution «National Medical Research Center of Endocrinology» of the Ministry of Health of Russia). In addition, all schoolchildren underwent an ultrasound examination of the thyroid gland (using a portable ultrasound machine LOGIQe (China) with a multifrequency linear transducer 10–15 MHz, in the supine position). The height and weight of children was determined according to the standard method at the time of the examination. A collection of samples of edible salt, which is used in families of schoolchildren, was carried out and the presence of iodine in it was determined by an express method for the qualitative determination of potassium iodate.

Parents of schoolchildren signed informed consent for the examination of children. Permission of the local ethical committee of the Endocrinology Research Centre — received, date: March 25, 2020, N 5.

**Results:**

Results: 227 schoolchildren of 8–10 years old were examined. The median concentration of iodine in urine was determined, the presence of iodine in food salt was investigated, and an ultrasound examination of the thyroid gland was carried out in order to clarify the iodine supply, the coverage of the use of iodized salt in nutrition and the prevalence of goiter.

The median concentration of iodine in urine was 153 μg/l, the frequency of goiter was 7.7%, and the proportion of households using iodized salt was 95.2%.

**Conclusion:**

Conclusion: Results of assessment (median urinary iodine concentration) confirm that population of Tuva, Russian Federation, has optimum iodine nutrition. The prevalence of goiter in schoolchildren significantly decreased compared to earlier assessments. The proportion of households using iodized salt indicates the effectiveness of preventive measures in the ­region.

## ОБОСНОВАНИЕ И КРАТКИЙ ОБЗОР ПРОБЛЕМЫ

К йододефицитным заболеваниям (ЙДЗ) относят все патологические состояния, развивающиеся в популяции в результате дефицита йода в питании, которые могут быть предотвращены при нормальном потреблении йода (определение ВОЗ) [1][2]. Самым распространенным проявлением ЙДЗ является диффузный нетоксический зоб. Существует четкая зависимость между низким содержанием йода в пище и воде и развитием зоба у населения. Когда йод поступает в достаточном количестве, наблюдается значительное уменьшение распространенности зоба [3]. Хронический дефицит йода приводит также к развитию узлового зоба, умственной и физической отсталости детей, невынашиванию беременности [1–6]. ЙДЗ определяют состояние здоровья населения и интеллектуальный уровень общества [7]. Согласно заключению ВОЗ, недостаточность йода является самой распространенной причиной умственной отсталости, которую можно предупредить [8].

Еще в 1915 г. Дэвидом Марином были сформулированы современные методы профилактики зоба как самого распространенного проявления дефицита йода [2]. В том же году в Швейцарии Ханцигер предложил использовать йодированную соль для профилактики зоба. Наиболее эффективным и безопасным методом решения проблемы дефицита йода в популяции является йодирование пищевой поваренной соли, производимое путем добавления к обычной соли йодата калия [2].

В 1990–2006 гг. сотрудниками ЭНЦ РАМН (в настоящее время «НМИЦ эндокринологии» МЗ РФ) были проведены широкомасштабные эпидемиологические исследования, результаты которых показали, что в Российской Федерации практически нет регионов, где население не подвергалось бы риску развития ЙДЗ. Йодный дефицит был в большей степени выражен в восточной части страны по сравнению с западной. В некоторых удаленных регионах были обнаружены тяжелые проявления йодного дефицита [2].

Мониторинг эффективности профилактических программ осуществляется путем организации непрерывной оценки обеспеченности населения йодом. Критериями эффективности программ массовой йодной профилактики, рекомендованными ВОЗ и ЮНИСЕФ, являются значение медианной концентрации йода в моче, доля домохозяйств, использующих в питании йодированную соль, и распространенность эндемического зоба у школьников [8, 9]. Причем основным эпидемиологическим показателем, характеризующим обеспеченность йодом жителей того или иного региона на данный момент времени, является медианная концентрация йода в моче. Данный показатель считается высокочувствительным, быстро реагирующим на изменения в уровне потребления йода, имеет важнейшее значение не только для оценки эпидемиологической ситуации, но и для осуществления контроля программ профилактики ЙДЗ. Выбор репрезентативной группы для оценки йодной обеспеченности популяции осуществляется кластерным методом, что является наиболее эффективным и обоснованным с практической точки зрения [2]. Чаще всего подобные исследования проводятся на базе школ, включают детей 8–10 лет. Другой эпидемиологический показатель состояния йодного дефицита — распространенность зоба — в большей степени отражает результаты долгосрочных мероприятий, а не текущую ситуацию [8][9].

В 1996–1998 гг. в Республике Тыва впервые было проведено комплексное эпидемиологическое исследование с целью оценки тяжести дефицита йода и распространенности ЙДЗ с использованием современных методов, по результатам которого был выявлен тяжелый йодный дефицит. Впервые обнаружен очаг тяжелой йодной недостаточности с высокой распространенностью ЙДЗ: в Чаа-Хольском районе республики медианная концентрация йода в моче (мКЙМ) составила 16,1 мкг/л, экскреция йода с мочой была снижена у 98,5% взрослых и у 100% детей; распространенность зоба по данным пальпации щитовидной железы (ЩЖ) у школьников составила 83,4%, по данным ультразвукового исследования (УЗИ) — от 27,2 до 98,5% в разных возрастных группах. Были выявлены случаи эндемического кретинизма и проведено изучение его клинико-эпидемиологических особенностей совместно с профессором G. Robert DeLong (Duke University Medical School, Durham, NC, USA). Частота повышения тиреотропного гормона (ТТГ) при неонатальном скрининге (более 5 мЕд/л) по республике составила 37,6%, что соответствовало тяжелому йодному дефициту. В западной, наиболее населенной части республики неонатальный ТТГ выше 5 мЕд/л определялся в различных районах с частотой от 40,4 до 74,6% случаев. Распространенность эндемического кретинизма в республике достигала 3,5%, при этом были выявлены различные его формы: неврологический, смешанный, микседематозный, с преобладанием последнего (77,6%), имеющего характерные признаки тяжелого гипотиреоза, умственной отсталости, эмоционально-волевых нарушений. По результатам проведенных исследований были даны рекомендации по обязательному использованию йодированной соли в питании населения на постоянной основе как средства массовой профилактики ЙДЗ [10].

В 1998–1999 гг. Правительством Республики Тыва совместно с учеными НИИ медицинских проблем Севера СО РАМН разработана программа ликвидации йодного дефицита «Неотложные меры борьбы с йододефицитными заболеваниями в Республике Тыва на 2000–2002 годы», основанная на использовании йодированной поваренной соли и препаратов йода (антиструмина и йодида калия) для массовой и групповой профилактики. Мероприятия в рамках данной программы фактически стали реализовываться с 1999 г., практически с момента ее разработки. В районы (кожууны) была завезена йодированная соль, школьникам, беременным и кормящим женщинам стали назначать антиструмин с содержанием йода в одной таблетке 1000 мкг.

В последующие годы проводился мониторинг ЙДЗ и эффективности профилактических мероприятий. Так, исследования концентрации йода в моче у школьников, проведенные в 1998–2001 гг., позволили оценить динамику йодной обеспеченности на фоне проведения йодной профилактики в республике. Например, в конце 1998 г. в западном Сут-Хольском районе мКЙМ составляла 26,6 мкг/л, 80% обследованных детей препубертатного возраста имели уровни экскреции йода с мочой менее 50 мкг/л и 28% — менее 20 мкг/л, что свидетельствовало о недостаточной эффективности профилактических мероприятий в этом районе, причем аналогичные данные были получены и в ряде других районов республики. Однако уже в 1999 г., на фоне начала активных профилактических мер, частота значений неонатального ТТГ >20 мЕд/л уменьшилась в целом по республике в 4,5 раза (c 8,48 до 1,76%). Также в 1999 г. в целом по республике в 12,7 раза снизилась частота значений неонатального ТТГ от 50 до 100 мЕд/л, а значения ТТТ более 100 мЕд/л не выявлялись [11].

По данным исследования 2000 г., проведенного в отдаленных и труднодоступных районах Тывы — Тоджинском и Каа-Хемском, сохранялась недостаточная йодная обеспеченность населения: мКЙМ у детей составила 56,1 мкг/л и 38,2 мкг/л соответственно. Однако в это же время в г. Кызыл уже выявлялась нормальная йодная обеспеченность школьников, регулярно получавших антиструмин: мКЙМ составила 181,4 мкг/л, при этом у 79% обследованных КЙМ превышала 100 мкг/л и только у 5% была менее 50 мкг/л, что свидетельствовало об адекватности йодной профилактики. Исследования, проведенные в 2001 г. в западных — Чаа-Хольском, Дзун-Хемчикском районах и на юге Тывы — в Овюрском районе, показали, что мКЙМ у детей препубертатного возраста соответствовала целевым значениям. Более 50% проб мочи имели концентрацию йода от 100 до 200 мкг/л и менее 20% проб — менее 50 мкг/л.

В 2000–2001 гг. ЭНЦ РАМН совместно с Министерством здравоохранения республики организована экспедиция в 5 кожуунов с целью проведения мониторинга зобной эндемии на фоне йодной профилактики, по итогам которой отмечена положительная динамика: частота зоба снизилась до 30–50%, мКЙМ повысилась до 90–100 мкг/л. Было рекомендовано продолжить проведение индивидуальной йодной профилактики препаратами йодида калия беременным и кормящим женщинам, широко использовать йодированную соль в питании населения.

По данным анкетирования населения Республики Тыва в 2008 г., проведенного под руководством главного внештатного эндокринолога Чубаровой Р.В., пищевая поваренная йодированная соль употреблялась уже в 47% домохозяйств, всем беременным в женских консультациях назначались препараты йодида калия. Тем не менее, несмотря на проводимую профилактику, по данным официальной статистики, за 2012–2015 гг. отмечался некоторый рост заболеваемости эндемическим зобом среди подростков: с 18 380 в 2012 г. до 21 630 в 2015 г., что послужило основанием для более активной разъяснительной работы с населением со стороны врачей-эндокринологов и терапевтов. В 2016 г. данный показатель несколько улучшился — было выявлено 14 183 случаев, однако в целом сохранялась высокая заболеваемость ЙДЗ — 3532,9 на 100 тыс. населения, что было на 82% выше среднероссийских показателей; причем наиболее высокая заболеваемость отмечалась у детей и подростков (в 4,5 и 4 раза выше, чем в среднем по РФ).

В 2016 г. Правительство Республики Тыва издало распоряжение «Об утверждении межведомственного плана мероприятий по формированию здорового образа жизни у населения Республики Тыва на 2016–2018 годы» [12], согласно которому, предприятиям пищевой и перерабатывающей промышленности рекомендовано использовать йодированную соль при производстве молочной продукции и хлебобулочных изделий; управлению Роспотребнадзора и Министерству сельского хозяйства и продовольствия поручено проводить контрольные мероприятия по использованию йодированной соли при производстве продуктов питания. Данные мероприятия существенно повлияли на обеспеченность населения республики йодом.

Не исключено, что реализация этих мер стала причиной позитивных изменений, зафиксированных Росстатом, а именно: за период 2017–2019 гг. общая заболеваемость патологиями ЩЖ в Республике Тыва составила 3361,8 случая на 100 тыс. населения, в том числе на долю заболеваний, связанных с дефицитом йода, приходилось 2811,2 случая. По результатам неонатального скрининга заболеваемость врожденным гипотиреозом составила всего 2 случая на 6989 новорожденных в 2017 г., в 2018–2019 гг. случаев врожденного гипотиреоза зафиксировано не было [13].

Таким образом, результаты неонатального скрининга подтверждают эффективность профилактических программ: по данным медико-генетической службы республики, в 2019 г. при обследовании 6023 новорожденных только у 128 (2,1%) уровень ТТГ превышал 5 мЕд/л.

В настоящее время основной акцент в регионе сделан на массовой йодной профилактике путем использования йодированной соли в питании населения.

С целью оценки эффективности профилактических мероприятий в регионе ФГБУ «НМИЦ эндокринологии» МЗ РФ по поручению Минздрава России в октябре 2020 г. проведено эпидемиологическое исследование, направленное на уточнение текущей йодной обеспеченности населения.

## ЦЕЛЬ ИССЛЕДОВАНИЯ

Оценка текущей йодной обеспеченности населения Республики Тыва.

## МАТЕРИАЛЫ И МЕТОДЫ

Исследование проводилось в Республике Тыва: в центральной части — в г. Кызыл; г. Шагонар (Улуг-Хемский кожуун), находящемся в 115 км западнее г. Кызыл, и п.г.т. Сарыг-Сеп (Каа-Хемский кожуун), расположенном в 90 км к юго-востоку от столицы.

В исследование были включены ученики следующих средних общеобразовательных учреждений: в г. Кызыл — МБОУ «Гимназия №5» и МБОУ «Гимназия №9», в г. Шагонар — МБОУ СОШ №1, в Каа-Хемском кожууне — МОУ «СОШ №1 им. Ю.А. Гагарина» п.г.т. Сарыг-Сеп, МОУ «СОШ №2 им. С.К. Тока» п.г.т. Сарыг-Сеп, МБОУ СОШ п. Усть-Бурен, МБОУ СОШ с. Бурен-Хем, МБОУ СОШ с. Дергиз-Аксы. 

Исследование проводилось одномоментно с 12 по 16 октября 2020 г.

Выбор школ и населенных пунктов проводился с учетом количества обучающихся детей и возможности обследовать одномоментно не менее 30 детей в возрасте 8–10 лет.

Выборка обследованных детей была сформирована методом систематического выбора с учетом обучения в школах не только детей, проживающих в данном городе или поселке, но и детей, приезжающих из других населенных пунктов региона.

Дизайн исследования — одномоментное популяционное.

## МЕТОДЫ

Обследовано 227 школьников допубертатного возраста (8–10 лет) (рис. 1) с проведением сбора анамнеза, осмотра врача-эндокринолога, пальпации ЩЖ, забора разовых образцов мочи в одноразовые микропробирки типа Эппендорф, с последующей заморозкой до -20–25°С. Рост и вес детей определялись по стандартной методике в момент обследования.

**Figure:**
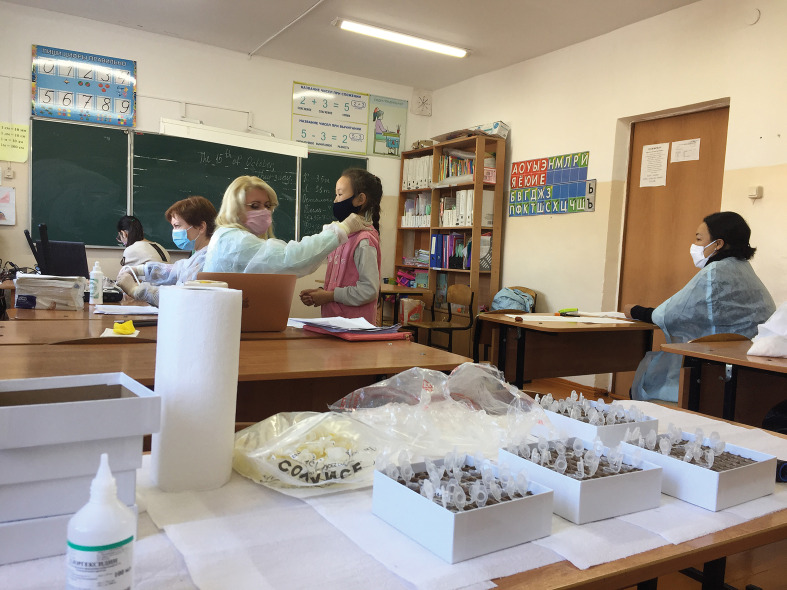
Рисунок 1. Сотрудники ФГБУ «НМИЦ эндокринологии» проводят обследование в Республике Тыва.

Всем школьникам выполнено ультразвуковое исследование (УЗИ) ЩЖ. УЗИ ЩЖ проводилось в соответствии с рекомендациями ВОЗ с использованием портативного ультразвукового аппарата LOGIQe (China) с мультичастотным линейным датчиком 10–15 МГц в положении лежа. Объем ЩЖ рассчитывался по формуле:

Vщж = [(Шпр. × Дпр. × Тпр.) + (Шл. × Дл. × Тл.)] × 0,479,

где Vщж — объем щитовидной железы;

Шпр. — ширина правой доли щитовидной железы;

Шл. — ширина левой доли щитовидной железы;

Дпр. — длина правой доли щитовидной железы;

Дл. — длина левой доли щитовидной железы;

Тпр. — толщина правой доли щитовидной железы;

Тл. — толщина левой доли щитовидной железы.

У детей соответствие объема ЩЖ нормативным показателям, разработанным Zimmermann М. и соавт., оценивалось с учетом площади поверхности тела и пола [14].

Концентрация йода в моче определялась церий-арсенитным методом в клинико-диагностической лаборатории ФГБУ «НМИЦ эндокринологии» Минздрава России. Данная методика обеспечивает выполнение измерений концентрации общего йода в моче в диапазоне концентраций 20–300 мкг/л (0,16–2,37 мкмоль/л). Общий йод представляет собой сумму связанного йода и свободного йодида [15].

Родители школьников подписали информированные согласия на проведение обследования детей. Разрешение локального этического комитета ФГБУ «НМИЦ эндокринологии» Минздрава России получено 25 марта 2020 г., № 5.

Был проведен сбор образцов пищевой соли, которая используется в семьях школьников. 213 образцов были исследованы на предмет содержания йодата калия с использованием экспресс-метода.

Принцип метода заключается в изменении окраски раствора крахмала при выделении свободного йода из соли после обработки ее тест-раствором. Степень изменения окраски оценивается визуально.

## Статистический анализ

Данные представлены в виде абсолютных значений и процентов от общего количества. Для описательного статистического анализа концентрации йода в моче были использованы значения медианы и частотного распределения.

## Этическая экспертиза

Протокол исследования одобрен на заседании этического комитета ФГБУ «НМИЦ эндокринологии» Минздрава России от 25 марта 2020 г. (протокол № 5).

## РЕЗУЛЬТАТЫ

мКЙМ у школьников составила 153 мкг/л, что соответствует целевым значениям и подтверждает достаточное его потребление. Частотное распределение показателей концентрации йода в моче в обследованной выборке представлено на рисунке 2.

**Figure:**
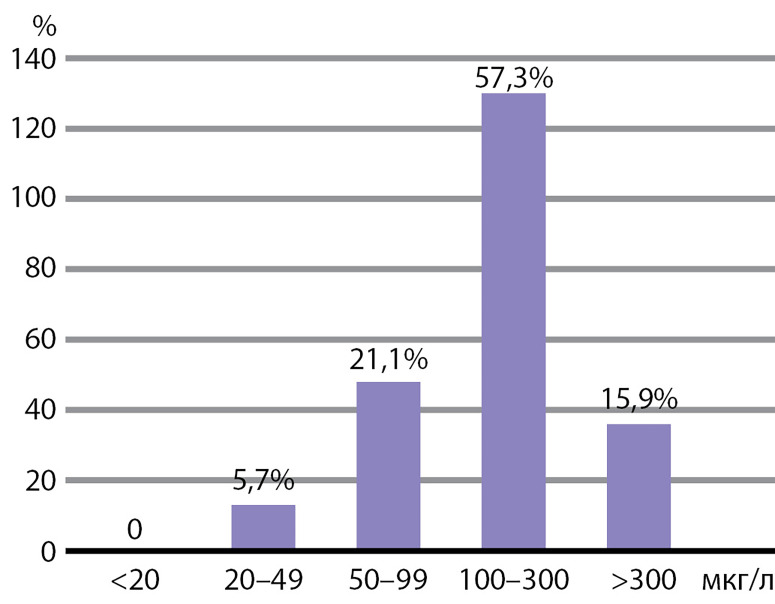
Рисунок 2. Частотное распределение концентрации йода в моче у обследованных школьников.

Частота зоба у детей по данным УЗИ варьировала от 3,3 до 11,1%. Средняя частота зоба в обследованной выборке составила 7,7%.

Результаты проведенных исследований в населенных пунктах Республики Тыва представлены в таблице 1.

**Table:** Таблица 1. Результаты исследований в населенных пунктах Республики Тыва

Населенный пункт	Распространенность зоба, %	Медианная концентрация йода в моче, мкг/л	Доля йодированной соли, %
г. Кызыл	8,2	197	95,2
г. Шагонар	3,3	182	94,7
п. Сарыг-Сеп	11,1	121	94,5

Во всех районах, в которых проводилось исследование, доля образцов йодированной соли составила практически 95% (рис. 3), что также соответствует критериям ВОЗ эффективности массовой йодной профилактики (не менее 90%).

**Figure:**
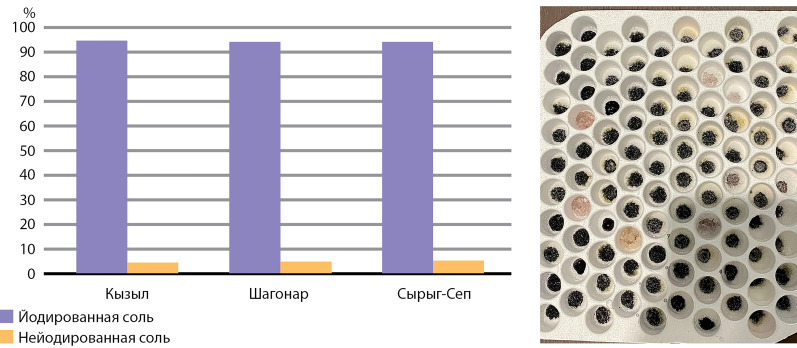
Рисунок 3. Результаты определения наличия йода в пищевой соли из домохозяйств (синее окрашивание образцов соли подтверждает наличие йодата калия).

Обращает на себя внимание, что более 90% всей пищевой соли, реализуемой населению в Республике Тыва, является йодированной. Органами здравоохранения на постоянной основе проводится активная разъяснительная работа о необходимости потребления исключительно йодированной соли.

Действительно, одним из ключевых компонентов профилактических программ по борьбе с ЙДЗ является информирование населения, прежде всего работниками здравоохранения. Примерами таких источников информации являются плакаты, разработанные медицинскими специалистами и размещенные на территории поликлиник республики (рис. 4).

**Figure:**
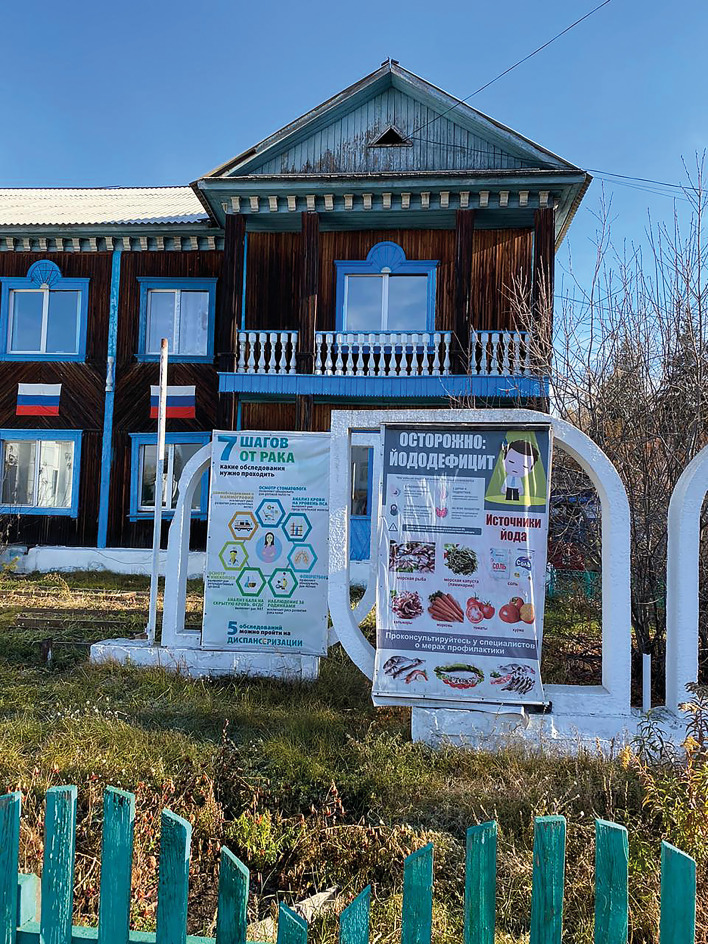
Рисунок 4. Поликлиника п. Сарыг-Сеп, информационные плакаты для населения.

При сравнении показателей, характеризующих йодную обеспеченность населения прошлых лет, с показателями 2017–2020 гг. можно отчетливо проследить динамику от тяжелого йодного дефицита до адекватной обеспеченности населения йодом, что, бесспорно, связано с использованием йодированной соли большей частью населения Республики Тыва.

## ОБСУЖДЕНИЕ

Целью проведенного исследования было оценить эффективность массовой йодной профилактики в Республике Тыва. При планировании исследования нами учитывались методические рекомендации ВОЗ, ЮНИСЕФ, IGN, в качестве оцениваемых параметров были выбраны три основных индикатора — мКЙМ, доля домохозяйств, использующих йодированную соль в питании, и частота зоба у школьников.

Результаты проведенного исследования позволяют говорить об адекватной йодной обеспеченности населения республики, поскольку и мКЙМ, и доля домохозяйств, использующих йодированную соль, соответствуют целевым значениям. Следует отметить, что именно значение мКЙМ наиболее быстро меняется на фоне проводимых профилактических мероприятий.

ВОЗ декларирует, что в условиях нормальной йодной обеспеченности частота зоба у детей школьного возраста должна быть не выше 5%. Следует учитывать, что после нормализации потребления йода потребуется несколько лет, прежде чем частота зоба у школьников станет ниже 5%. Очевидно, что этот показатель не может измениться быстро. Ожидать снижения распространенности зоба можно через 5–10 лет, в течение которых профилактические мероприятия будут проводиться на постоянной основе. Тем не менее уже сейчас впечатляет достигнутый результат: распространенность зоба у школьников за последние 20 лет снизилась в 12 раз и составляет всего лишь 7,7%.

Помимо того, что частота зоба может считаться лишь косвенным показателем уровня потребления йода, на показатели распространенности зоба, по данным УЗИ, влияет ряд объективных и субъективных факторов, включая квалификацию специалиста, технические погрешности при изменении размеров железы и другие факторы. У ребенка объем ЩЖ зависит от степени физического развития, поэтому полученный показатель сопоставляется с нормативами в зависимости от площади поверхности тела, полученными в регионах без дефицита йода. Референсные значения будут зависеть и от этнического состава обследованной когорты детей. В настоящее время общепринятых стандартов объема ЩЖ у детей нет.

Здравоохранение Республики Тыва уверенно демонстрирует успех в решении проблемы тяжелого дефицита йода, последовательно проводя профилактические мероприятия в регионе, что подтверждается результатами проведенного нами исследования.

Результаты исследования свидетельствуют о том, что в Республике Тыва (регионе с тяжелым природным йодным дефицитом) в настоящее время имеет место достаточная обеспеченность населения йодом. Медианная концентрация йода в моче составляет 153 мкг/л, что, согласно рекомендациям ВОЗ, отражает адекватное йодное обеспечение и прямо коррелирует с использованием йодированной соли в домохозяйствах (более 90%).

Распространенность диффузного нетоксического (эндемического) зоба у школьников достоверно снизилась с 96% в 1996–1998 гг. до 7,7% в 2020 г. на фоне массовой йодной профилактики йодированной солью.

## Сопоставление с другими публикациями

Сравнивая полученные нами результаты с другими подобными исследованиями, стоит отметить опыт Республики Беларусь, где на постоянной основе проводится массовая йодная профилактика. Йодный дефицит в республике был ликвидирован (мКЙМ, по данным многочисленных исследований, выше 100 мкг/л) благодаря принятию в 2001 г. Постановления Правительства РБ об обязательном использовании йодированной соли при производстве продуктов питания [16]. В последующем, в 2008 г. эти меры были закреплены в дополнении к Закону Беларуси «О качестве и безопасности продовольственного сырья и пищевых продуктов для жизни и здоровья человека» [17].

Аналогичная картина по устойчивому устранению дефицита йода в питании наблюдается во многих странах, которые ввели массовую йодную профилактику йодированной солью, например, в Армении, где начиная с 1997 г. при технической поддержке Детского фонда ООН (ЮНИСЕФ) было возобновлено производство йодированной соли, а выпуск нейодированной соли для населения и пищевой промышленности был прекращен. В 2004 г. в Армении принято Постановление Правительства, согласно которому вся производимая в стране пищевая поваренная соль должна быть йодированной, а импорт нейодированной соли запрещен [18]. Йодный дефицит в стране был ликвидирован, что подтверждается результатами национальных эпидемиологических исследований [18].

## Клиническая значимость результатов

Клиническая значимость полученных нами результатов состоит в подтверждении эффективности программы массовой йодной профилактики с использованием йодированной соли, результатом осуществления которой стало значительное снижение распространенности диффузного нетоксического зоба.

## Ограничения исследования

В Республике Тыва неоднократно проводились подобные исследования [1][2][6][11]. Ограничением нашего исследования является недостаточно широкий охват населения удаленных и труднодоступных районов. Тем не менее, по нашему мнению, выборка достаточно репрезентативна с учетом предшествующих обследований. В дальнейшем планируются проведение мониторинга ситуации в регионе, обследование жителей труднодоступных районов, в т.ч. в рамках разрабатываемой региональной программы профилактики ЙДЗ на последующие годы.

## Направления дальнейших исследований

В продолжение работы планируются исследование йодной обеспеченности беременных и кормящих женщин на фоне массовой йодной профилактики, уточнение и научное обоснование необходимости дополнительной йодной дотации в виде лекарственных препаратов йода данным группам населения.

## ЗАКЛЮЧЕНИЕ

Аналитический обзор ситуации с йодной обеспеченностью населения Республики Тыва и динамики распространенности сопряженной с дефицитом йода патологии, в первую очередь зоба у школьников, а также актуальные данные, полученные в ходе контрольно-эпидемиологического исследования 2020 г., убедительно продемонстрировали эффективность массовой йодной профилактики йодированной солью. Пример Республики Тыва наглядно иллюстрирует возможность устранения тяжелого дефицита йода и достижения целевых значений экскреции йода с мочой при помощи системного потребления домохозяйствами исключительно йодированной соли и является весомым аргументом в пользу принятия в России закона о профилактике ЙДЗ при помощи йодированной соли. Разработка и реализация региональных профилактических программ, в т.ч. направленных на проведение системного биологического мониторинга эффективности йодной профилактики, — станут абсолютно гармонизированным шагом в контексте профилактики на государственном уровне.
